# Analysis of anions in beer using ion chromatography

**DOI:** 10.1155/S1463924602000172

**Published:** 2002

**Authors:** Jonathan Bruce

**Affiliations:** Metrohm UK Ltd Metrohm House, Unit 2 Top Angel Buckingham Industrial Park Buckingham MK18 1TH UK

## Abstract

The majority of anions found in beer are a consequence of impurities derived from the water used during the brewing process. The process of beer manufacture consists of malting, brewing and fermentation followed by maturation before filtration and finally storage. Strict quality control is required because the presence of certain anions outside strictly defined tolerance limits can affect the flavour characteristics of the finished product. The anions present were quantified using the technique of ion chromatography with the Metrohm modular system following sample preparation. The analysis produced a result of the order 200 mg l^-1^ for chloride, phosphate and sulphate and around 20 mg l^-1^ for nitrate. If the chloride level exceeds 250 mg l^-1^, then the sweetness of the beer is enhanced, but yeast flocculation can be hindered. An excess of sulphate can give a sharp, dry edge to hopped beers and excessive amounts of nitrate have been found to harm the yeast metabolism after conversion to the nitrite form. As water is a primary ingredient within beer, its quality and type is a fundamental factor in establishing many of the distinctive regional beers that can be found in the United Kingdom and is thus monitored carefully.


Journal of Automated Methods & Management in Chemistry
Vol. 24, No. 4 (July–August 2002) pp. 127–130

Taylor & Francis

Taylor & Francis Group

### Analysis of anions in beer using ion chromatography

**Jonathan Bruce**
*Metrohm UK Ltd, Metrohm House, Unit 2, Top Angel, Buckingham Industrial Park, Buckingham MK18 1TH, UK*

*The majority of anions found in beer are a consequence of impurities derived from the water used during the brewing process. The process of beer manufacture consists of malting, brewing and fermentation followed by maturation before filtration and finally storage. Strict quality control is required because the presence of certain anions outside strictly defined tolerance limits can affect the flavour characteristics of the finished product. The anions present were quantified using the technique of ion chromatography with the Metrohm modular system following sample preparation. The analysis produced a result of the order 200 mg $l^{-1}$ for chloride, phosphate and sulphate and around 20 mg $l^{-1}$ for nitrate. If the chloride level exceeds 250 mg $l^{-1}$, then the sweetness of the beer is enhanced, but yeast flocculation can be hindered. An excess of sulphate can give a sharp, dry edge to hopped beers and excessive amounts of nitrate have been found to harm the yeast metabolism after conversion to the nitrite form. As water is a primary ingredient within beer, its quality and type is a fundamental factor in establishing many of the distinctive regional beers that can be found in the United Kingdom and is thus monitored carefully.*

### Introduction

The UK is one of the world's top brewing nations, beaten only by the USA, China, Germany, Japan and Brazil. It is estimated that British brewers brew more than 34 million barrels a year, with the public house being the destination that most people choose to consume their beer. More than 1200 different beers are brewed in the UK, with a staggering 28 million pints being consumed each day.

Beer consists of four primary ingredients: barley malt, which gives the beer its fullness, hops, which gives the beer its bitterness, yeast, which coverts the barley into alcohol, and, water, which assists the fermentation and distillation processes before reducing the finished product into consumable proofs.

The majority of anions found in beer are a consequence of the water used during the brewing process and strict quality control is required because their presence can affect the flavour of the finished product. An excessive amount of chloride and sulphate can have an adverse affect on the flavour of the beer. High concentrations of nitrate can also pose a problem if they are converted to the nitrite form, which can harm the yeast metabolism leading to incomplete fermentation.

As well as assaying the finished beer product, ion chromatography (IC) can also be used to quantify the intermediate raw materials used in the brewing process like water to ensure purity and consistency. Water is one of the primary ingredients used and still many arguments remain unresolved among the different brewers about whether soft or hard water produces a better-finished product. Hard waters will contain higher amounts of mineral salts and sulphates, all of which can be analysed using the technique of IC.

### Origins of beer

The exact origins of beer are unknown. The Greek historian Herodotus cited the Egyptians as making the first true beer. Other more recent evidence suggests that the Sumerians were the first beer drinkers some 10 000 years BC. Whichever supposition is right, the one fact that remains clear is that beer has been around for an extremely long time. Ale was brewed for many centuries without the use of hops; instead, mild herbs like rosemary were used for flavouring. The first hops-flavoured beers were introduced into England sometime during the 15th century. Before the advent of refrigeration in the 1880s, beer was actually brewed only during the colder months of September to April.

### Manufacture of beer

The basic process of beer manufacture consists of malting, brewing and fermentation followed by maturation, filtering and bottling. To make beer, the water and barley are used to create a sweetened liquid called wort, which is flavoured with hops before being fermented with yeast.

The practice known as malting prepares the barley for use in the brewing process as the grain is naturally dry and hard and cannot be used in its common state. The starch in the floury kernel of barley is insoluble in water so the grain is steeped in water before being spread on racks until rootlets appear. The germination process produces enzymes that break down the insoluble starches in the grain converting them to sugars. When the shoots reach a certain length, the barley (now known as green malt) is dried in a kiln at 50 °C to stop the germination process. The temperature is then elevated to about 85 °C for light malt—or higher for dark malt—before the shoots are removed (milling) and the dried malt stored in silos. Some beer producers also add unmalted rice or wheat along with the malted barley to give the beer a different subtle flavour.

The procedure of turning the ground malt into the sweet liquid wort is called brewing. The milled barley is mixed with warm water then gradually heated to around 75 °C in large pots (tuns) where the starch in the mash is

 © 2002 Taylor & Francis Ltd
http://www.tandf.co.uk/journals
DOI: 10.1080/14639240210159049

converted into various sugars. The used grains are filtered out of the tun and hops are added, which give the beer its aroma and bitter taste as well as preserving the mixture. The wort is usually boiled for 1–2 hours to extract the essence from the hops and sterilize it before cooling takes place using a heat exchanger. The wort product is then saturated with air in readiness for fermentation of the yeast.

Yeast is a single-cell, microscopic, living organism that is part of the fungus family. The addition of yeast converts the sugars in the wort into alcohol and carbon dioxide as well as a range of subtle flavours. The process of fermentation takes between 5 and 12 days and can occur at varying temperatures depending on the type of beer being created. Different breweries possess assorted strains of yeast, most of which are kept a closely guarded secret, and these largely determine the character of the beer. In some yeast varieties, the cells rise to the top of the beer mixture by the end of fermentation process before being skimmed off. When the yeast has done its job, the head settles into a thick creamy crust that protects the beer from air. This technique is known as top fermentation and ales are brewed in this manner. Conversely, when the yeast cells sink to the bottom, this is known as bottom fermentation and it is the method used for brewing of lagers or pils. For lagers, the yeast tends to work at cooler temperatures.

The beer has now been brewed, but its taste is enhanced through maturation where the fermented wort is set aside at near freezing temperatures to mature for several days (at least), which allows the beer to develop flavour as the beer ripens removing the rough edges. The conditioning undertaken depends on how the beer leaves the brewery.

For cask-conditioned beers (real ales), the beer goes directly into a cask, barrel or bottle where additional hops may be added for extra aroma. Finings are added that bind the materials responsible for haze and sink to the bottom clarifying the beer. The yeast in the beer is still active and the beer undergoes a second fermentation in the cask, usually in the cellar of the pub. Cask conditioned beer is a delicate product and unfortunately is vulnerable to attack from contamination by strains of wild yeast and other biological organisms. Thus, great care is required to protect the integrity and character of the beer.

Other types of beers are conditioned in the brewery. Some are fined and filtered with carbonation, others are pasteurized to prevent deterioration from microbes extending the shelf life of the product before it reaches the consumer usually in kegs, cans or bottles. Lagers require a longer period of conditioning in the brewery at lower temperatures—the word 'lager' comes from the German *lagern*, meaning to store at a cold temperature. Most modern breweries use an automated process where the bottles are filled, capped and labelled at a rate of up to 100 000 bottles per hour. Purists suggest that modern manufacturing processes rob the beer of some of its natural flavours, such is the price one pays for an extended product shelf-life and the chance to discover different imported and regional beers.

### Ion chromatography: an overview

Chromatography is a method for separating mixtures of substances using two phases, one of which is stationary, the other mobile moving in a particular direction. Chromatography techniques are divided according to the physical states of the two participating phases. The term 'ion exchange chromatography' or 'ion chromatography' (IC) is a subdivision of high-performance liquid chromatography (HPLC).

A general definition of IC can be applied as follows: 'ion chromatography includes all rapid liquid chromatography separations of ions in columns coupled online with detection and quantification in a flow-through detector'.

A stoichiometric chemical reaction occurs between ions in a solution and a solid substance carrying functional groups that can fix ions because of electrostatic forces. In anion chromatography, these are quaternary ammonium groups. In theory, ions with the same charge can be exchanged completely and reversibly between the two phases. The process of ion exchange leads to a condition of equilibrium, the side to which the equilibrium lies depends on the affinity of the participating ions to the functional groups of the stationary phases.

### Analysis of beer

A total of 50 ml beer was poured into a clean beaker. The beer was then decanted to another clean beaker to facilitate the removal of carbon dioxide. The process was repeated 20 times before sonication for 15 min. The beer was then diluted 1:20 with deionized water. Of the diluted beer sample, 20 $\mu$l were injected directly into the ion chromatograph and the response for the peaks recorded using a mobile phase eluent of sodium carbonate/sodium bicarbonate with the Metrohm A SUPP 5 analytical column. The calculation was carried out automatically using integration software IC Net 2.1 against a previously prepared calibration plot.

The modular system used for the analysis of beer comprised the Metrohm 709 IC Pump, Metrohm 732 IC Detector, Metrohm 733 IC Separation Center, Metrohm 752 Pump Unit and Metrohm 762 IC interface.

The 709 IC pump comprises a dual piston pump with two valves to guarantee low residual pulsation and excellent flow stability. The pressure was measured extremely precisely by piezo-resistive means, which allows the facility to set the pressure upper and lower limits to safeguard the pressure-sensitive columns.

The 732 IC detector comprises a detector block with a built-in measuring cell that is thermally and electrically shielded from outside influences with the use of a Faraday cage. The measuring cell is thermostatted and has a thermal stability of $< 0.01\,^{\circ}$C. Together with the electronic preamplification in the detector block, one can obtain very high sensitivity coupled with an optimal signal-to-noise ratio. The liquid-contacting parts and the electronic parts are separated from each other.

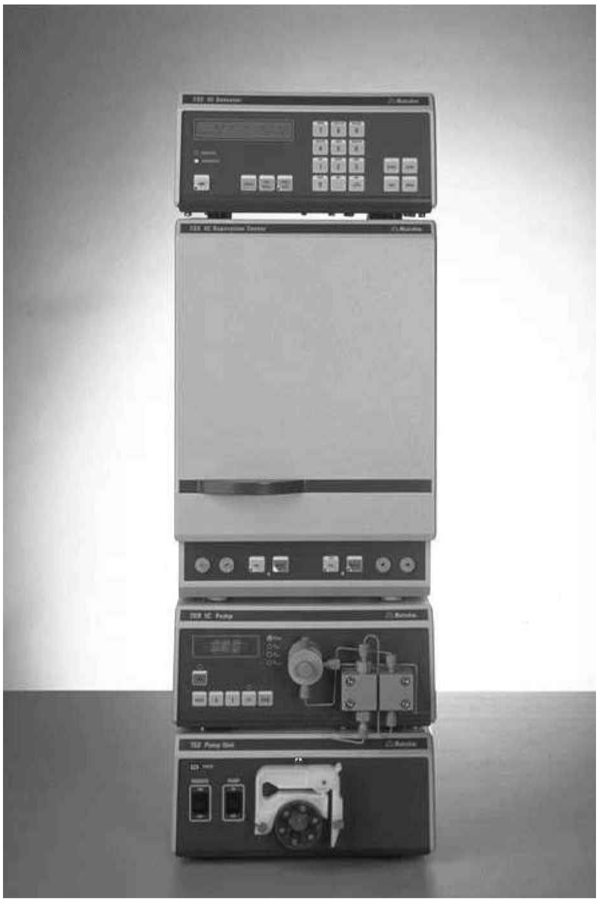

*Figure 1. Modular system used for beer analysis.*

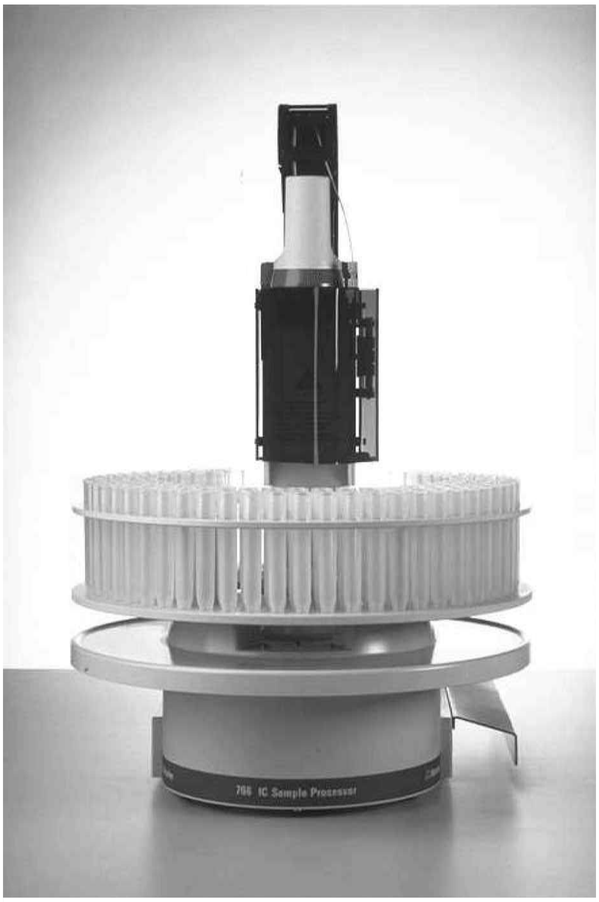

*Figure 2. 766 IC Sample Processor.*

The 733 IC separation centre provides thermal stability and the metal network built into the casing provides an effective shield against electromagnetic radiation. The separation centre is the home of all the components associated with the wet chemistry part of IC analysis; guard columns, analytical columns, detector cell, pulsation dampner, Metrohm Suppressor Module (MSM) and valves all work under steady and constant ambient conditions.

The MSM is built into the IC separation centre in certain instrument configurations and consists of three microbed-packed suppressor channels located inside a rotor. One channel is used inline for the analysis, with the other two operating offline. A fresh suppressor channel is used each time for analysis ensuring that the cation exchanger is operating at full capacity. The advantages of this type of suppressor are that there are no sensitive membranes, no contamination by sulphuric acid, no risk of destruction by pressure or heavy metals and no hydrogen gas production in the laboratory.

The 752 IC pump unit is used with the MSM supplying the two offline channels; one is sulphuric acid for regeneration, the other deionized water for rinsing.

With the 762 IC interface and Metrohm IC Net software, complete control of the Metrohm hardware is guaranteed under a Windows operating system. The IC interface is responsible for the data acquisition and analogue-to-digital conversion of the outputted signal from the detector. It is possible to control up to 16 IC or HPLC peripheral devices through the interface with only a single connection to the personal computer. The IC Net software allows full control of all individual instruments in any given system through the use of pictographic icons as well as performing all the important integration functions.

When a large sample throughput is required, it is recommended that one automate the system with one of three Metrohm autosamplers: 766 IC Sample Processor, 788 IC Filtration Sample Processor or the 813 Compact Autosampler.

The analysis produced a result of approximately 200 mg $l^{-1}$ for chloride, phosphate and sulphate, and 20 mg $l^{-1}$ for nitrate.

### Conclusion

Monitoring the anion profile is an important quality-control step in the brewing industry. If the level of chloride is > 250 mg $l^{-1}$, then this level can enhance the sweetness of the beer but it hinders yeast flocculation, so the level needs to be carefully observed. An excess of sulphate gives a sharp, dry edge to well-hopped beers and the level present should be minimized as much as possible bearing in mind that sulphate occurs naturally in water.

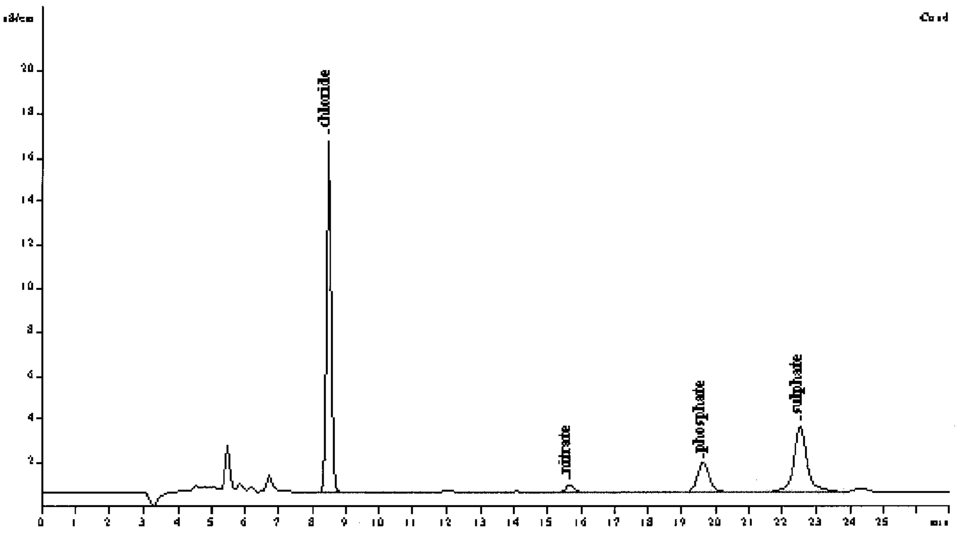

*Figure 3. Chromatograph for a sample of beer.*

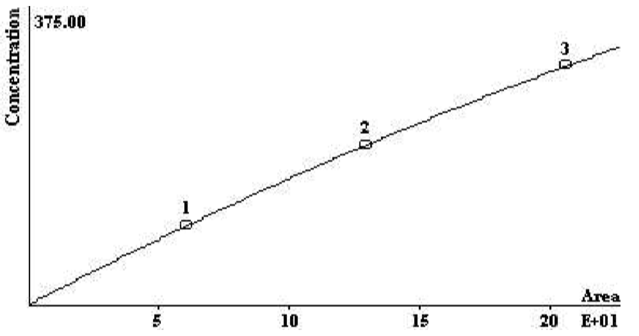

*Figure 4. Calibration plot for the chloride component for a sample of beer.*

Phosphate is present in the malt and buffers the mash to a slightly acidic pH. Quantifying the level of nitrate is important as excessive amounts can hamper the fermentation process after conversion to nitrite. The quality and type of water is a fundamental factor in establishing many of the distinctive regional beers that can be found in the UK.

The finished beer product and intermediates used throughout such as the brewing water can be easily determined using IC to ensure manufacturing authenticity. One of the major advantages of IC as an analytical technique is that often little sample preparation is required and needs only a small amount of sample. The running costs of IC with Metrohm instruments are surprisingly low, only requiring the acquisition of chemicals necessary for the eluent and suppressor module as well as a clean, reliable supply of deionized water.

### References

The following Internet sites were used extensively as references and can be accessed for further information:

http:\\www.beerandpub.com
http:\\www.beerstuff.com
http:\\www.interbrew.com
http:\\www.metrohm.ch
http:\\www.stellartois.co.uk

